# Student biocuration projects as a learning environment

**DOI:** 10.12688/f1000research.72808.2

**Published:** 2022-02-02

**Authors:** Katherine E. Thurlow, Ruth C. Lovering, Sandra De Miranda Pinheiro

**Affiliations:** 1 Functional Gene Annotation, Preclinical and Fundamental Science, Institute of Cardiovascular Science, University College London (UCL), London, WC1E 6JF, UK

**Keywords:** Biocuration, Gene Ontology, community curation, student gene annotation

## Abstract

**Background:** Bioinformatics is becoming an essential tool for the majority of biological and biomedical researchers. Although bioinformatics data is exploited by academic and industrial researchers, limited focus is on teaching this area to undergraduates, postgraduates and senior scientists. Many scientists are developing their own expertise without formal training and often without appreciating the source of the data they are reliant upon. Some universities do provide courses on a variety of bioinformatics resources and tools, a few also provide biocuration projects, during which students submit data to annotation resources.

**Methods:** To assess the usefulness and enjoyability of annotation projects a survey was sent to University College London (UCL) students who have undertaken Gene Ontology biocuration projects.

**Results:** Analysis of survey responses suggest that these projects provide students with an opportunity not only to learn about bioinformatics resources but also to improve their literature analysis, presentation and writing skills.

**Conclusion:** Biocuration student projects provide valuable annotations as well as enabling students to develop a variety of skills relevant to their future careers. It is also hoped that, as future scientists, these students will critically assess their own manuscripts and ensure that these are written with the biocurators of the future in mind.

## Introduction

Bioinformatics is utilised in a wide range of scientific disciplines, including data science and statistics as well as the biological sciences. ^1^ As such, a research project in bioinformatics requires the incorporation of skills from a multitude of areas and can be utilised in a plethora of future careers, such as computational biologists, bench scientists, project managers, copywriters, clinicians, bioinformaticians or administrators. Recent technological advancements in high-throughput research have resulted in vast amounts of data output, which is outstripping analysis capabilities. ^2^ Therefore, a priority for bioinformatics research is to increase efficiency and the rate at which peer-reviewed experimental data is captured.

### Biocuration as a teaching tool

Biocuration is the process of capturing biological knowledge in a database, usually through the creation of annotations. Typically, this process involves an in-depth review of the published literature and then, using standardised terms and identifiers, condensing the information into a computer-readable format. This process has many similarities to a literature review project that many students undertake during their undergraduate and postgraduate studies. The main difference being that at the end of a literature review the student must provide a well-written summary of their investigations, whereas a biocurator needs to learn and apply knowledgebase specific annotation rules to summarise the reviewed data.

Since the turn of the century and launch of the postgenomic era it has become more apparent that bioinformatics has the potential to improve the research efficiency of biologists, biomedical researchers, statisticians and clinicians alike. With the wide range of bioinformatics resources to choose from, finding and using the gold standard resources can be challenging. Consequently, many universities now offer bioinformatics courses to undergraduates, postgraduates and staff. While many of these courses focus on providing training on specific resources, such as Ensembl, ^3^ UniProt ^4^ or functional analysis tools, ^5- 7^ a few universities, including University College London (UCL), encourage students to contribute annotations to public biological knowledgebases. ^8- 12^ For example, over the past 10 years the Functional Gene Annotation group (UCL) has provided a 10-week bioinformatics MSc module as well as a Gene Ontology biocuration project to undergraduate and postgraduate students.

### Gene Ontology

**Figure 1.**
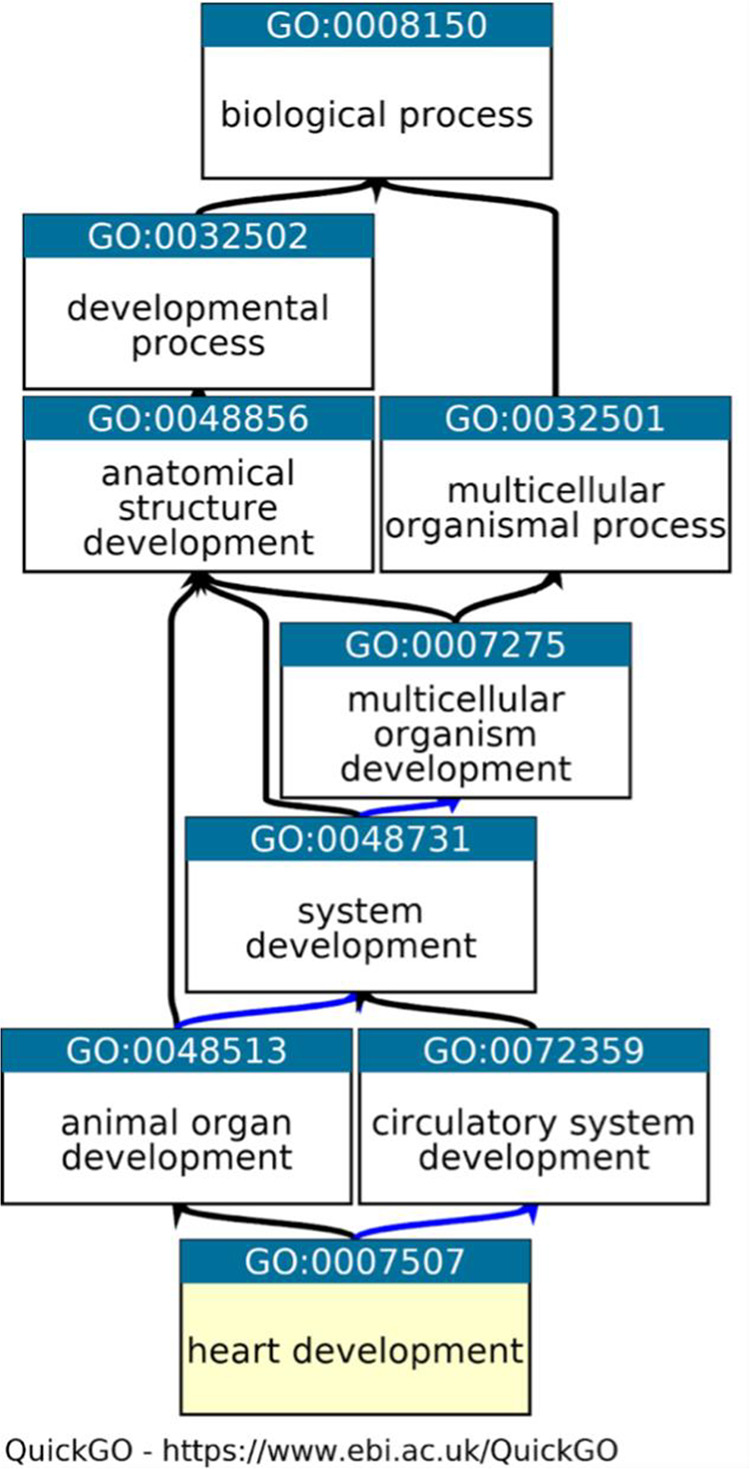
A fragment of the Gene Ontology. A fragment of GO, representing the relationships between the GO term ‘heart development’ (highlighted in yellow) and its parent, less specific GO terms. Graph downloaded from QuickGO, ^44^ black arrows indicate is_a relations between terms and blue arrows indicate part_of relations ( https://www.ebi.ac.uk/QuickGO/term/GO:0007507) Access date: 19/05/2021.

The aim of the Gene Ontology (GO) is to capture the vast amount of published biological and biomedical data describing gene products into an accessible and computer-readable format. ^13^^,^^14^ The GO knowledgebase is split into two primary intertwining sections. Firstly, the ontology provides summary statements (GO terms) that describe the molecular functions (e.g. enzyme activity), biological processes (e.g. immune response) and cellular locations (e.g. cytoplasm) of gene products, with hierarchical and directional relationships between GO terms ( Figure 1). Secondly, GO annotations link specific gene or gene product identifiers to the ontology terms, thereby placing the gene products within a biological context and allowing a statement to be made about their functional role and location in the cell. The standardisation of language through GO terms supports accurate curation. A variety of approaches are taken to create GO annotations, ranging from manual curation, by biocurators, based on information presented in peer-reviewed published literature ( Table 1), through to automatic pipelines, extracting data from bioinformatics databases, such as InterPro and Reactome. ^15- 17^ There is an additional novel, third aspect to GO; GO-Causal Activity Models (GO-CAMs), which compiles GO annotations into comprehensive gene function models and can include causal inferences between molecular activities. ^18^ This feature of GO was not available for any of the projects within this study.

**Table 1.** Selection of manual annotations associated with hsa-miR-153b-3p. Two annotations, created during a UCL GO biocuration project, associating human miR-153b-3p with molecular function and biological process GO terms. These annotations were supported by a luciferase assay ^45^ and, therefore, assigned a direct assay evidence code. ^14^ The annotation extension field enables the biocurator to capture the miRNA target, APP (UniProtKB ID: P05067) and the tissue (brain) in which these activities and processes are occuring in. Data downloaded from QuickGO, ^44^ access date: 21/05/2021.

RNA central ID	URS0000068B85_9606	
miRNA Name	hsa-miR-153b-3p	
Qualifier	enables	involved_in
GO term	GO:0003730	GO:0035195
GO name	3′-UTR mRNA binding	gene silencing by miRNA
ECO ID	ECO:0000314 (IDA, inferred from direct assay)	
Reference	PMID:22733824	
Taxon ID	9606	
Assigned by	ARUK-UCL	
Annotation Extension	has_input (UniProtKB:P05067, APP) part_of (GO:0035195, gene silencing by miRNA) Occurs_in(UBERON:0000955, brain)	has_input (UniProtKB:P05067, APP) Occurs_in(UBERON:0000955, brain)
GO aspect	Molecular Function	Biological Process

### Importance of GO

The GO has been cited by over 100,000 publications as of 2020, ^18^ demonstrating its value and global use. The increasing quantity of high-throughput data analysis being conducted in recent years has meant annotation of genomes and proteomes is of particular importance, as the quality of gene annotations impacts on the accuracy of data interpretation. The GO is being used in Genome Wide Association Studies, to increase the likelihood of identifying disease-risk genes through the grouping of genes involved in the same disease associated pathway. ^19^^,^^20^ Furthermore, GO is used for biomarker identification, ^21^ the identification and further elucidation of risk predictions, ^22^ as well as to interpret transcriptomic and proteomic datasets associated with systemic diseases affecting multiple systems, such as cancers. ^23- 25^ Thus, current research projects are becoming more reliant on computationally accessible GO annotation data to identify gene products that could be used as either potential drug targets, or as prognostic or diagnostic biomarkers. GO annotations extensions ^26^ also allow for elucidation of cellular and tissue context for gene function or involvement in particular processes ( Table 1), which can also inform research with drug repurposing or assessment of off-target effects.

The interpretation of high-throughput datasets can be hampered by the researcher’s lack of understanding of the underlying resources used in the analysis. Therefore, increasing education in bioinformatics will provide researchers the knowledge and skills to appropriately interpret their data. Provision of sufficient bioinformatics training has long been a challenge, ^27- 29^ which is being partially addressed globally with 62 events on the TeSS Bioinformatics training platform ^30^ (access date: 02/06/21) and over 100 open-source training materials and resources for bioinformatics available from the GOBLET training portal ^31^ (access date: 02/06/21), many of which are available directly from the resource providers. However, while the TeSS platform includes events covering data mining and machine learning, only a handful of these events are specifically for biologists and clinicians using bioinformatics. Furthermore, there is a lack of biocurator focused courses. ^27- 29^

### GO biocuration projects

The primary objective for a UCL student undertaking a GO biocuration project is to annotate within a theme to complete a thesis. Consequently, these projects involve in depth reading of scientific articles surrounding a disease-related process or set of genes. For example, recent project titles have focused on curating microRNA regulation of junctional proteins at the blood-brain barrier and amyloid processing in the context of Alzheimer’s disease, as well as the role of specific signalling pathways in heart development ( https://www.ucl.ac.uk/cardiovascular/student-projects). The GO annotations created by the students are checked by a professional biocurator and submitted to the GO Consortium knowledgebase. As the Functional Gene Annotation group has been previously funded by the British Heart Foundation, Alzheimer’s Research UK and Parkinson’s UK, the projects have primarily surrounded cardiovascular and neurological disease-associated processes or gene sets.

**Figure 2.**
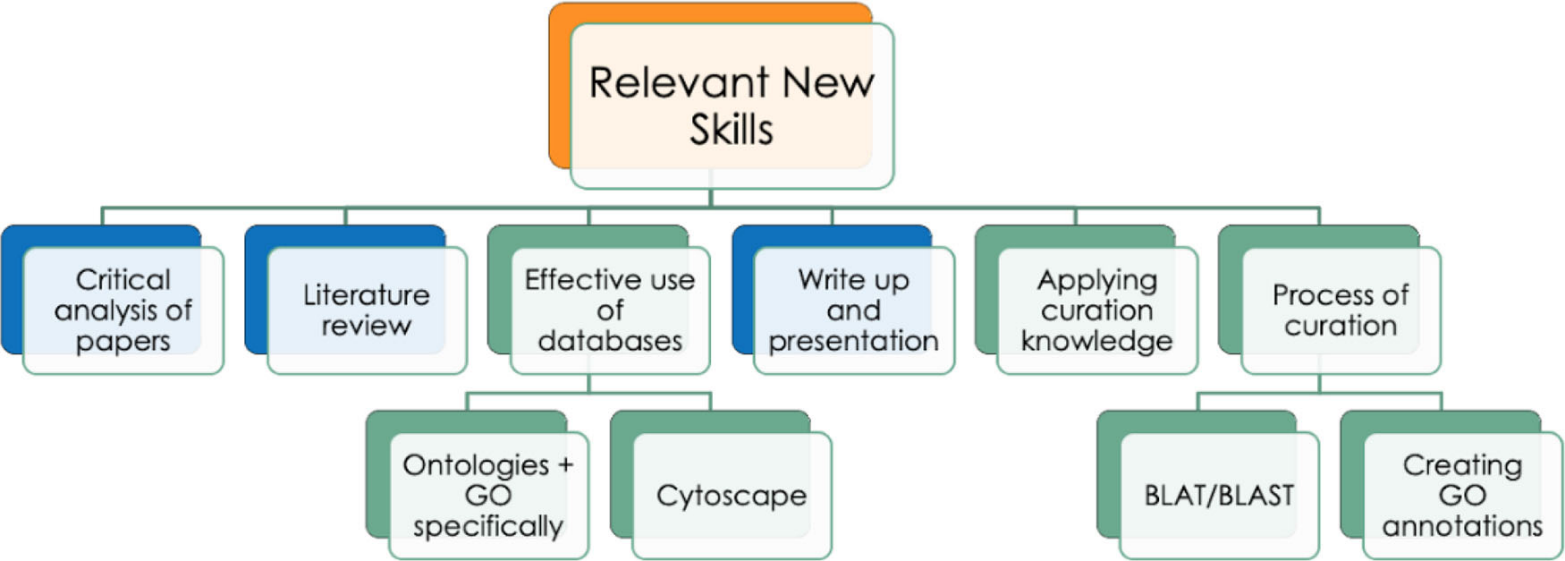
Outline of relevant new skills students can expect to obtain through a GO biocuration project. The skills highlighted in blue are general project skills which are common to undergraduate and graduate dissertations. The skills highlighted in green are specific to the GO biocuration project. Although, some, or all, of these skills may also be gained in other types of research projects.

There are three primary overarching learning objectives for a GO biocuration project, firstly, to improve the student’s understanding of experimental methods through critical analysis of articles identified through a literature search. Secondly, for the student to gain extensive experience of using bioinformatics resources in concert, applying theoretical knowledge to practical utilization. Finally, to increase the student’s understanding of ontologies and the process of manual annotation. Secondary learning outcomes include improving the student’s knowledge surrounding the biological context of the project, gaining confidence in GO analysis, as well as a change in the student’s approach to both general research and bioinformatics research in particular. Many of the projects carried out as part of one of the degrees represented in this study also require the student to complete a 10,000 word dissertation and to provide a 10-15 minute project presentation. For the purpose of this study, the skills students can expect to gain were split into those that would be acquired during the majority of UCL student projects, such as writing a dissertation and giving a project presentation, and skills specific to the GO biocuration projects, such as the process of curation and use of bioinformatics databases and tools ( Figure 2).

**Figure 3.**
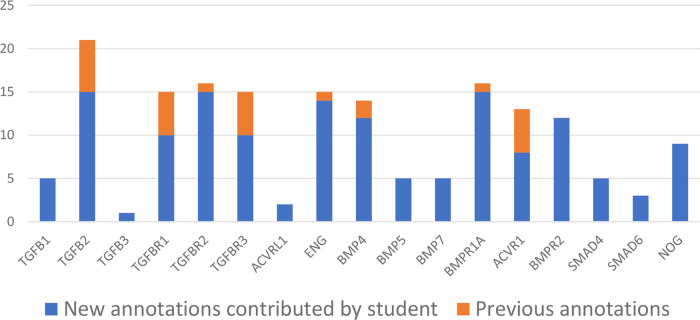
Impact of a student’s biocuration project on the GO Consortium knowledgebase. The histogram indicates the number of 'heart development' GO terms, associated with seventeen specific proteins, that existed before the start of the student’s project (orange) and contributed by the student (blue). This student’s project focused on the curation of proteins with a known role in heart development. At the end of the project the student had submitted over 500 GO annotations through the review of 25 articles and associated specific GO terms from the 'heart development' domain of the ontology with the prioritised proteins.

**Figure 4.**
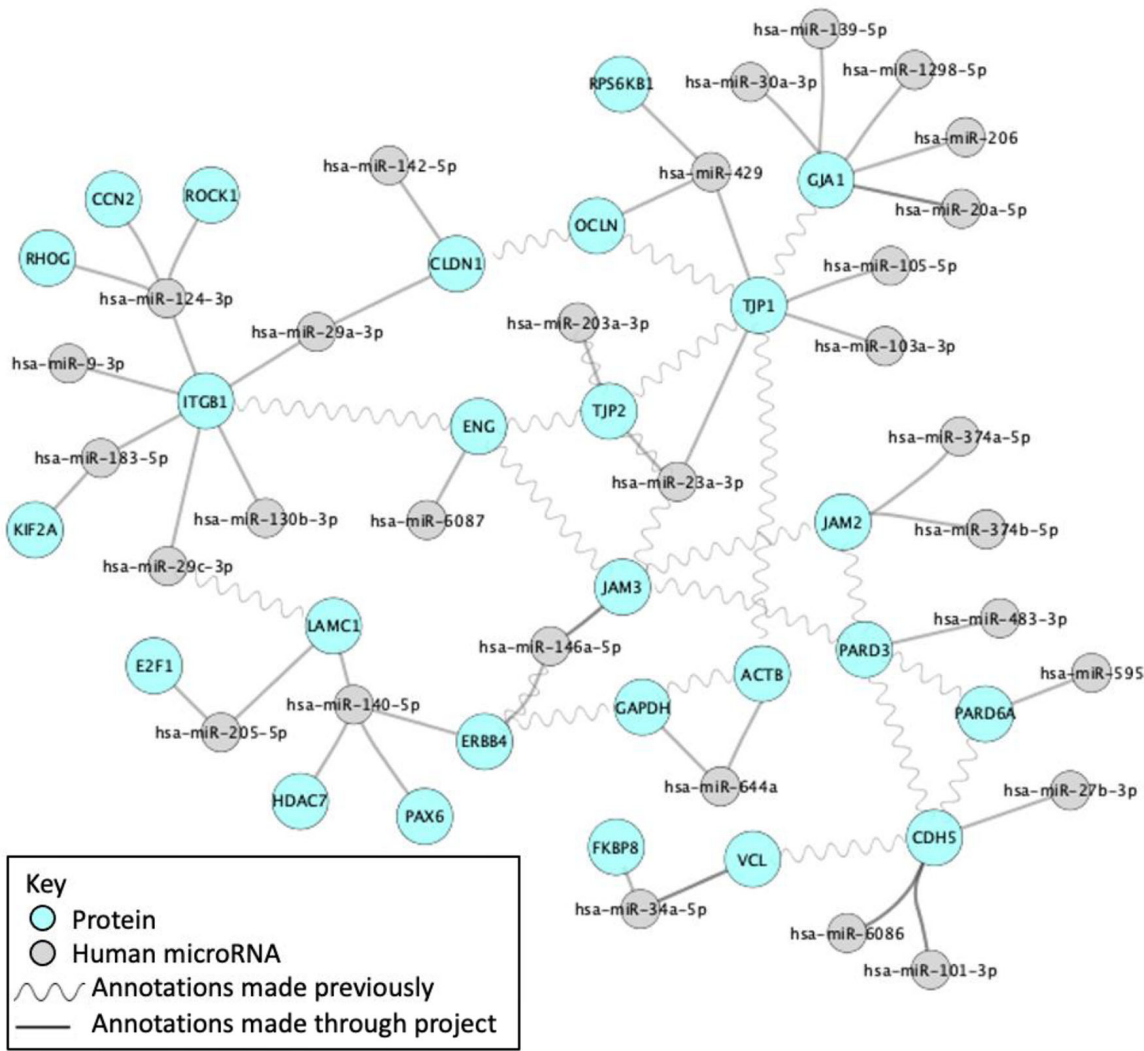
Interaction network created as part of a student GO biocuration project. This network was created using Cytoscape ^7^ and demonstrates the contribution of this project to an interaction network of blood-brain barrier-associated proteins and miRNAs.

Students will typically curate around 15-30 articles per project, requiring a good understanding of experimental methods, in addition to strong literature review and critical analysis skills. Each project usually leads to the creation of between 200 to 500 annotations associated with 35 to 55 gene products. To date, over 5,000 GO annotations have been associated with almost 900 entities, based on the review of over 500 articles by UCL students. As such, these student projects can substantially increase the coverage of the GO, through providing new GO annotations. For example, one project provided more detailed annotations describing the role of TGFB1, BMP5, ENG and BMPR1A in heart development ( Figure 3). In addition, the interactions captured through GO biocuration projects are included in the existing molecular interaction datasets ^32^ and can help elucidate relationships between interacting gene products ( Figure 4).

Students develop a range of transferable skills during the GO biocuration projects, that they would have developed when undertaking a bench or literature review project. For example, the students improve their writing and communication skills, their project and time management skills, as well as their ability to find and understand relevant data, critically evaluate this data and summarise the information. Student GO biocuration projects also have the potential to substantially increase a student’s skill-set, improving their ability to use bioinformatics resources to answer a research question, and increasing their understanding of experimental methods and ontologies. Thus, these students will be better placed to use biological and biomedical knowledgebases effectively in their future careers. Additionally, these projects have a secondary benefit to the wider biocuration community through a considerable contribution to the GO knowledgebase and the training of potential future expert biocurators and community biocurators. Another potential benefit, is that as future researchers these students may write future articles more sympathetically to biocuration, thus making data curation more efficient overall. This study aims to assess the usefulness and enjoyability of GO biocuration projects from the student perspective through analysis of quantitative and qualitative survey responses.

## Methods

The aim of this study was to consult past and present students to assess whether GO biocuration projects provide relevant and useful skills to students. A secondary research question addressed whether these projects lead to a wider appreciation of bioinformatics and its applications and also whether students would be interested in a community biocuration initiative.

### Participants and setting

An invitation to complete an open online questionnaire was circulated via email to 22 students who had either completed or were undertaking a GO biocuration project. In addition to specific questions about their views on the usefulness and enjoyability of various aspects of the project, demographic questions were collected. The first email inviting students to complete the survey was sent on 31 ^st^ March 2021. Reminders were automatically sent to students that did not complete the survey on 7 ^th^, 14 ^th^ and 20 ^th^ April, and the survey closed on 7 ^th^ May 2021.

All of the participants had undertaken a GO biocuration project, however, while the format for each project was generally the same, there was some variation. These variations were due to the study level of the student (which ranged from undergraduate to postgraduate), the interest of the student in learning key aspects of biocuration, the biological knowledge of the student, the title of the project, the project length (ranging from 4-8 months, full or part-time) and finally the timing of the project with respect to the Covid-19 pandemic. A list of the activities undertaken during the project are provided in Supplementary Table S10, *Extended data*. ^46^

All students chose a specific area of biology, usually linked to a specific disease, to curate. Some of the students focused on curating proteins, while others focused on microRNAs, but all students follow existing GO consortium guidelines. ^26^^,^^35^^,^^47^ For protein focused annotation projects, articles for curation are identified by a PubMed search using HUGO gene nomenclature (HGNC) approved symbols, ^48^ and key words (if there are more than 10 articles to review). Articles are only curated if there is sufficient information about the species of the protein used in the experiments and the article provides experimental data that describes the role of the protein in the disease or process that the student is curating. For microRNA projects the HGNC symbol of the microRNA target is used to search either miRTarBase ^49^ or emiRIT. ^50^ If no suitable articles are identified, then PubMed is searched with the HGNC symbol and key words such as microRNA (Supplementary Table S10, *Extended data* ^46^). Articles are only curated if there is evidence that the microRNA binds to the target mRNA that encodes for one of the prioritised proteins (usually this is provided by a reporter assay) and there is sufficient information about the species of the microRNA and mRNA used in the experiments. The publication date is not usually used to filter articles to curate.

### Survey design

The survey ( *Extended data:* Table S1A ^46^), created using opinio software ( https://opinio.ucl.ac.uk; version 7.12), was initially based around the Kirkpatrick model for assessment of training, ^33^ using three fundamental evaluation categories; reaction to training, learning outcomes and behaviour change, comprising 16 questions. Of these, 8 were considered demographic questions and 8 assessed the three evaluation categories. Quantitative metrics were obtained through the use of binary yes/no options or ratings ranging from 1-5. Comment boxes were included with several of the questions, to obtain qualitative data and supporting statements.

Demographic questions captured the year of study, the course undertaken while completing the project, the current role of the participant, whether the participant had previously attended a 10-week bioinformatics module, the participants view of their bioinformatics knowledge before and after the project, how frequently they use (or expect to use) bioinformatics resources and why they chose the GO biocuration project.

Reaction to training was assessed by asking the participants to rank the aspects of the project they found most useful to their personal development and to rank the aspects of the project they found most enjoyable.

Learning outcomes and behaviour change was investigated by asking the participants to rank the extent to which they agreed with five statements encompassing an improvement in overarching biological context knowledge, confidence in GO analysis interpretation, gaining unique skills and whether the way in which they conduct general and bioinformatics research had changed due to undertaking their project.

In addition, the participants were asked whether their views on the relevance/impact of bioinformatics had changed, whether they would recommend the project to future students and whether they would be interested in taking part in a community biocuration initiative.

### Qualitative analysis

The qualitative data was analysed using NVivo software version 12 and text condensation methodology, ^34^ as well as two independent researchers providing an overall impression of the data. Briefly, text condensation comprised thematic analysis of all qualitative responses (i.e., comment box content) where themes were characterised according to the research questions and project aspects. “Meaning units”, i.e., particular statements made by respondents, were identified from the text and coded to a particular theme. Repetition allowed for refinement and substantiation of the coding and clarification of themes. Condensation of these meaning units allowed for summation of the overall impression for each theme identified ( *Underlying data:* Table S9 ^46^) and collation of concepts to support the quantitative findings of the survey.

### Statistical analysis

Statistical analysis was conducted primarily on the reaction to training questions, but also to ascertain significance on learning outcomes and demographic questions including prior and post-project bioinformatics knowledge. Using Microsoft Excel for Mac, version 16.52, an F-test was performed to determine variance between; positive vs non-positive responses and then negative vs non-negative responses as well as between prior and post-project bioinformatics knowledge and bioinformatics module attendance ( *Extended data:* Table S4 ^46^). After determination of variance, t-tests were performed between the groups to ascertain significant difference ( *Extended data:* Table S5 ^46^). Lastly, Chi ^2^ tests were performed for all project aspects and learning outcomes to ascertain the significance of observed percentage responses compared to expected percentage responses ( *Extended data:* Table S6 ^46^). The expected percentage response for each rating 1-5 was 20%. ANOVA comparison of means was carried out to ascertain whether there was any correlation between attendance of the Genetics of Human Disease bioinformatics module and prior or post-project bioinformatics knowledge ( *Extended data:* Table S7 ^46^).

### Ethical considerations

Following UCL ethical guidelines, the research was classed as exempt from UCL ethical approval because the research involved the use of a non-sensitive, completely anonymous survey, the participants are not defined as “vulnerable”, and participation would not induce undue psychological stress or anxiety. Participants were informed in the invitation email and at the start of the survey that the submitted responses would be kept anonymous but that the data collected would be made public and that the feedback would be used to revise future annotation projects. Consent to participate in the study was implied by completion of the questionnaire. In addition, this article provides no information which would enable the answers to the survey to be linked to specific participant identities.

## Results

The survey examined the respondents view of their biocuration project with respect to a variety of aspects using both qualitative and quantitative questions. For the purpose of analysis, the questions were split up into demographic style questions and questions relevant to the biocuration project.

### Cohort summary

The survey was emailed to 22 past and present students who had undertaken GO biocuration projects with the Functional Gene Annotation group at UCL, with an 82% response rate and a 73% completion rate ( *Underlying data:* Table S1B ^46^). The demographic questions collected data on: the year the project was completed, stage of study, current job role, attendance of a 10-week UCL MSc bioinformatics module and current or expected frequency of use of bioinformatics resources ( *Underlying data:* Table S2 ^46^). Questions about the participants' view of their prior and post-project bioinformatics knowledge and the open question of why respondents chose a biocuration project were also included.

The majority (n = 13) of the respondents embarked on a biocuration project as part of an MSc qualification. Of the remaining respondents, three were iBSc students and two were PhD students. The survey cohort completed their projects over a span of ten years (2011-2021) with the majority of respondents completing their projects in 2019 or later (n = 11). The 18 respondents had a wide variety of current roles, including medical writer, clinical geneticist and post-doctoral fellow (Table S2). This provided an insight into how useful and relevant a GO biocuration project could be to areas of science that are not directly focused on bioinformatics or annotation, with one respondent stating: “ *I think bioinformatics is an essential skill to have and it can really help you with other areas too*” (respondent ID: 3440328).

**Figure 5.**
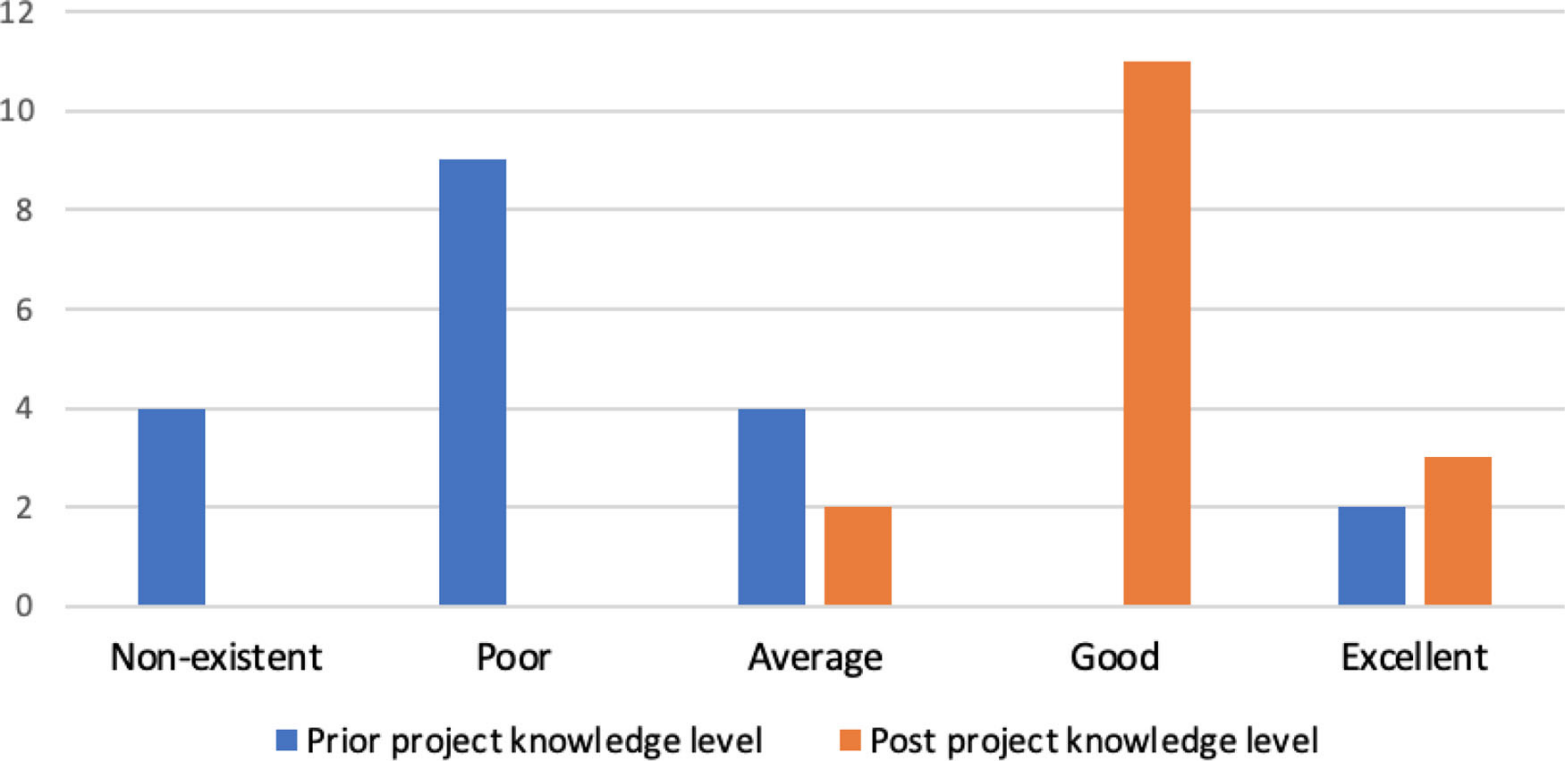
Self-rated bioinformatics knowledge of the respondents. The survey included two questions to investigate whether the student’s view of their own bioinformatics knowledge had changed after undertaking a biocuration project. Respondents rated the bioinformatics knowledge they had acquired before the start of the project (prior project knowledge level) and at the end of the project (post-project knowledge level) using ratings ranging from non-existent to excellent.

Over three quarters (77.8%) of respondents attended the 10-week UCL bioinformatics module, but this did not seem to correlate with either prior (t = 0.62, p-value = 0.27) or post-project (t = 1.34, p-value = 0.10) bioinformatics knowledge (Table S5). Although those that did attend the module had higher average scores of both prior (attended = 2.43, did not attend = 2.00) and post-project (attended = 4.17, did not attend = 3.67) bioinformatics knowledge, this difference was not statistically significant (p-value = 0.54 and 0.20 respectively from ANOVA comparison of means, Table S7). Almost all students (89.47%) stated that their understanding of bioinformatics was average or below at the start of their projects, with two respondents rating both their prior and post-project knowledge as ‘excellent’ ( Figure 5). Both of these individuals chose a biocuration project in order to broaden their skill-set and learn how to apply this knowledge to research, “ *I was keen to develop my skills in this area as I have always been interested in bioinformatics. I also wished to be involved in opportunities to expand this knowledge to real applications*” (3445442). Overall, respondents reported a significant improvement in bioinformatics knowledge after their project compared to before their project ( Figure 5, t = −5.43, p-value = 5.43 × 10 ^−6^) with 87.50% rating themselves above average (i.e. good or excellent).

**Figure 6.**
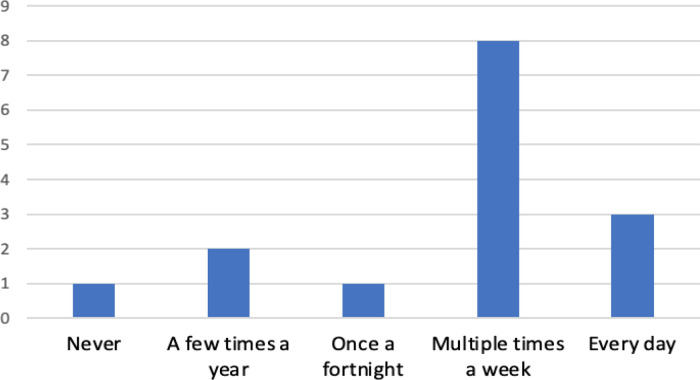
Current frequency of use of bioinformatics resources by respondents. Respondents were asked how frequently they use (or expect to use for current students) bioinformatics resources after completion of their project.

The majority of respondents (73.3%) either currently use, or expect to use, bioinformatics resources on a regular basis ( Figure 6). One respondent (3445928) stated: “ *I have used the resources a lot since. Also a lot of my PhD peers have never used these resources and always say how they wish they knew how to*”. Only one participant identified that they do not expect to use bioinformatics resources in the future, this individual is a current student and had to change their project plans due to COVID restrictions. Their response suggests that this student may be planning to follow either a laboratory-focused or non-research career path. The two ‘a few times a year’ responses were from a student and a medical writer, with the clinical geneticist, post-doctoral fellow and all of the PhD students, responding ‘multiple times a week’. All of the ‘everyday’ responses were from biocurators. The overall impression from our results is that the skills obtained through this project are highly transferable and that bioinformatics resources are relevant to a multitude of careers.

### Reasons for choosing project

There were a variety of reasons students initially chose a biocuration project, including the recent COVID pandemic restricting availability of laboratory-based projects and the overarching biological focus of the project e.g. Alzheimer’s Disease (Table S1B, Question 6). However, the three most common reasons were:

1. To gain new skills that would be useful in the future, “ *because I wanted to learn new skills and challenge myself. Before the project, I only had little experience with bioinformatics*” (3440328).
2. A preference for dry lab or computational work, “ *I was interested in improving my understanding of genetics, data science and paper interpretation as well as undertaking a non-lab based project*” (3440130).
3. Personal interest in bioinformatics, “ *It was an area that I wanted to explore as a potential career path*” (3445942).

The encompassing impression is that the majority of students undertook this project because they knew (or had been told) it would be useful, but not necessarily understanding why.

### Reaction to training

Quantitative analysis of both enjoyability and usefulness of project aspects as defined by the authors was carried out to assess the three primary learning outcomes of the projects. The aspects were split into general skills, such as carrying out a literature search, that could be expected to be gained by undertaking any research project that included a final dissertation and presentation, and specific bioinformatics skills, unique to a GO biocuration project, such as learning to use ontologies ( *Underlying data:* Table S3 ^46^). Respondents rated each aspect of their biocuration project from not at all useful or enjoyable to extremely useful or enjoyable. All responses were statistically significant with Chi ^2^ p-values ranging from 5.19 ×10 ^−9^ for enjoyability of presentation to 7.61 ×10 ^−49^ for usefulness of both critical evaluation of articles and write up (Table S6). The majority of our respondents (12 out of 18) did not report any negative experiences, and this view is supported by statements such as: *“(A) bioinformatics project is a good way to learn organizing information with a clear order and map their interactions.”* (3440087, Table S1B, Question 6). There were some negative responses ( *Underlying data:* Table S8 ^46^), with one participant stating that Cytoscape was ‘not at all’ useful or enjoyable and another that literature searches were not enjoyable. Presentations were considered by two participants as only slightly useful and by two as only slightly enjoyable. Only one participant gave a low score on several aspects. The student submitting the highest number of negative responses for this section considered GO annotation, databases, BLAT, BLAST and Cytoscape as ‘slightly useful’. As this respondent didn’t provide any comments from which we could have understood why they answered negatively for these particular aspects, but they did score enjoyability of these aspects higher.

*Usefulness and enjoyability of general skills*

A significant proportion (78.1%; t = 10.15; p-value = 3.9 × 10 ^−8^) of responses were positive about the general skills and tasks, either selecting considerably or extremely useful or enjoyable, and almost all responses (95.3%; t = 32.72; p-value = 6.3 × 10 ^−15^) were rated “non-negative” when including the neutral option “moderately”. The overall positive response scores for usefulness (96.7% non-negative) were higher than for enjoyability (93.3% non-negative).

**Figure 7.**
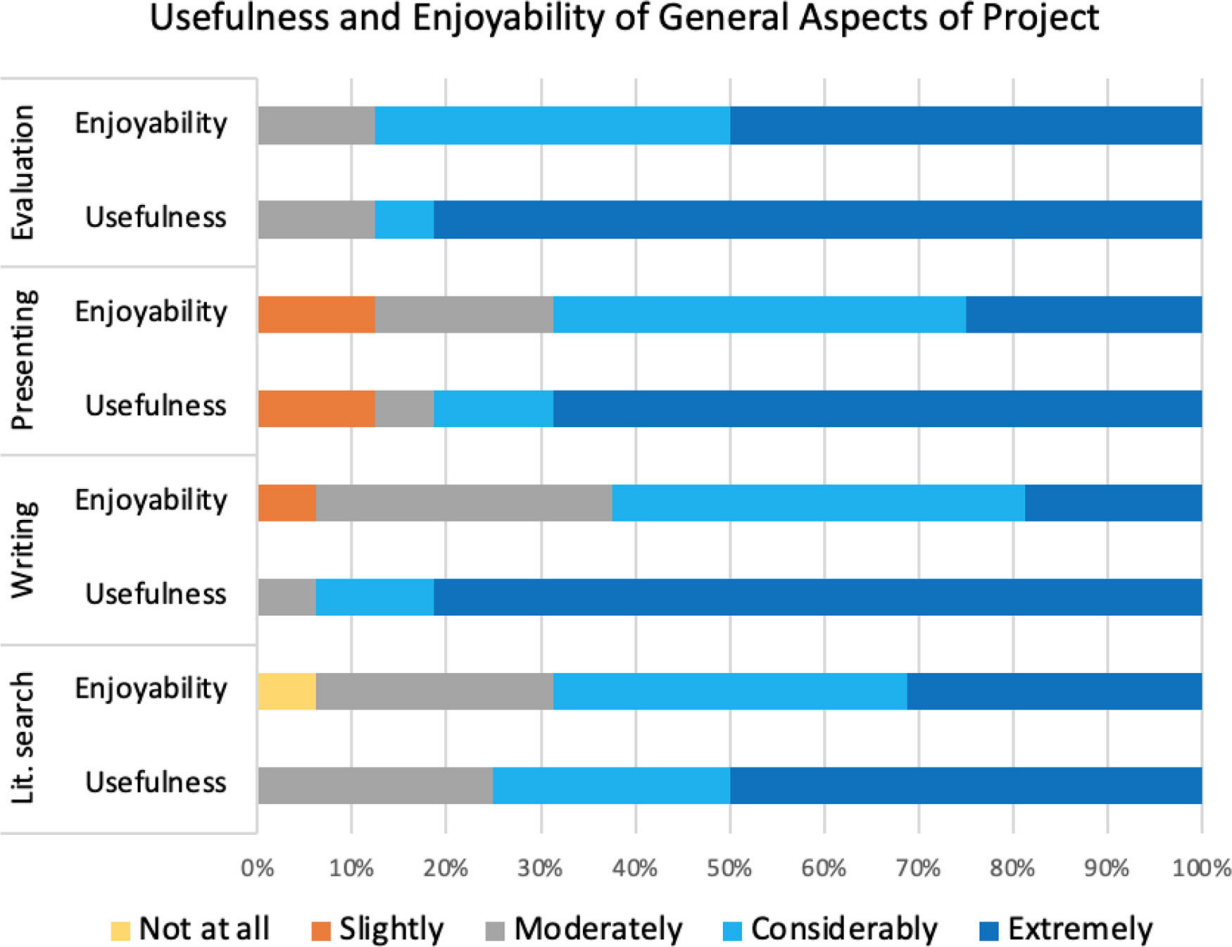
Respondents ranking of both usefulness and enjoyability of general project skills and tasks. The survey examined how the respondents ranked the usefulness of the skills they had gained through literature searching (lit. search), writing their dissertation (writing), presenting their project (presenting) and critically evaluating published articles (evaluation). In addition, the survey examined how much the respondents enjoyed these tasks. The ranking options range from “not at all” useful/enjoyable to “extremely” useful/enjoyable.

Like other student biocuration projects, the UCL student projects involved extensive searching of literature and critical evaluation of research, “ *It provided a set of tools that could be used as part of a literature search on an area of interest and an effective way to search for experimental data on a specific set of genes.*” (3445942), both of which will be vital skills for virtually any subsequent scientific career. ^21^ The survey confirmed that the majority of respondents (88%) particularly valued the critical evaluation skills they developed during their projects. The comments provided on this aspect of the biocuration project support this (Table S1B, Question 7, other specify), a typical response was *“this is something that is very useful not only for a career in scientific curation but many other scientific career paths”* (3440233). Almost all of the respondents considered that writing their dissertation was the most useful general aspects of the biocuration projects, with 95% rating this skill as considerably or extremely useful ( Figure 7). Fewer respondents indicated that the literature search aspect of the project provided them with useful skills (70% positive responses) although 100% of responses were non-negative.

This general agreement to the usefulness of these general skills, which would also have been developed during a laboratory-based project, also aligns with the qualitative findings. Over 30% of respondents identified critically analysing experimental data and literature searching as highly relevant skills to their current roles, with respondent 3440337 stating “ *There was a lot of searching literature and critical evaluation of research, both of which will be vital skills for virtually any subsequent career move in this field.*” Furthermore, respondent 3440130 mentioned *“Improving my ability to critically evaluate papers was crucial in my personal development as it allowed me to widen my understanding of different topics”* (Table S1B, Question 9). In general, the responses to the level of enjoyment of these tasks were similar to the views on the usefulness of these skills ( Figure 7) with only a 3.4% difference in non-negative responses.

*Usefulness and enjoyability of biocuration specific skills*

**Figure 8.**
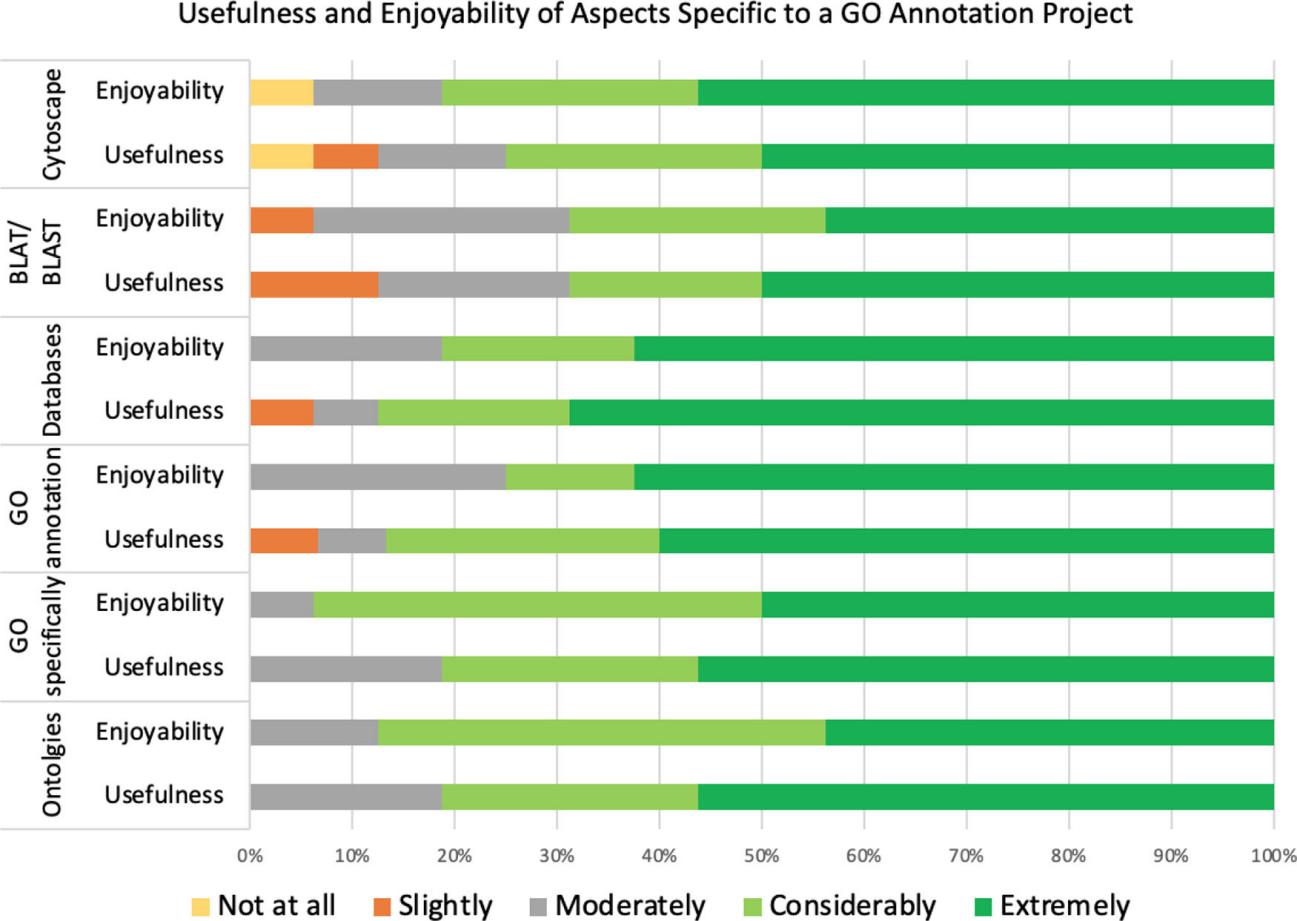
Respondents ranking of both usefulness and enjoyability of biocuration-specific project tasks. The survey examined how the respondents ranked the usefulness of understanding about and using ontologies and GO specifically, creating GO annotations, using a variety of databases and bioinformatics resources (Cytoscape, BLAT and BLAST). In addition, the survey examined how much the respondents enjoyed using these resources and creating GO annotations. The ranking options range from “not at all” useful/enjoyable to “extremely” useful/enjoyable.

The positive response score for usefulness (80%) was only slightly lower than for enjoyability (81.3%), with the overall biocuration specific aspects having a high rate of positive responses (80.6%; t = 19.62; p-value = 9.90 × 10 ^−16^). However, almost all responses (95.8%; t = 43.38; p-value = 4.14 × 10 ^−23^) were “non-negative”, when including the neutral option “moderately ( Figure 8).

When all responses concerning the usefulness of a biocuration project are compared, learning how to effectively use bioinformatics databases was ranked third most useful, after writing dissertation and critical evaluation of articles (Table S1B, Question 7). This was supported by respondents’ statements such as “ *I felt I learned a great deal … from learning about the existence of many different databases, to what they offered and how to use them and why*” (3432165). As expected, due to the wide range of current job roles of the respondents (Table S2), the positive responses for usefulness were slightly lower for the biocuration specific aspects of the project compared to the general aspects, with a difference of 4.6%. However positive responses for enjoyability were 8.2% higher for biocuration specific aspects than for the general aspects.

Although understanding ontologies may be regarded as a bioinformatics skill relevant to a broader range of scientific careers than GO annotation, GO annotation was found to be just as useful as learning about ontologies. One comment “ *Through both background literature searches and the annotation process of the project I came to realise how influential and valuable ontologies and annotations can be*” (3436438), suggests that the similar rating of GO annotation and ontologies is because the two are inextricably linked and therefore the process of annotation aids in the general understanding and appreciation of ontologies and their applications. Although the usefulness ratings were the same for GO and general ontologies, people generally enjoyed using GO more, suggesting the interface of GO is more user friendly, or that the frequent use of the GO browsers made these more enjoyable tasks than occasionally looking for other ontology terms. Alternatively, a student’s rating of the GO ontology as enjoyable may reflect the satisfaction of being able to see their annotations in the GO knowledgebase. The statement *“It was really satisfying to see my annotations get uploaded onto the databases”* (3440328) supports this idea (Table S1B, Question 9). Learning how to use the bioinformatics resources BLAT, BLAST and Cytoscape were generally found to be the least useful aspects of the projects.

### Learning outcomes and behaviour changes

**Figure 9.**
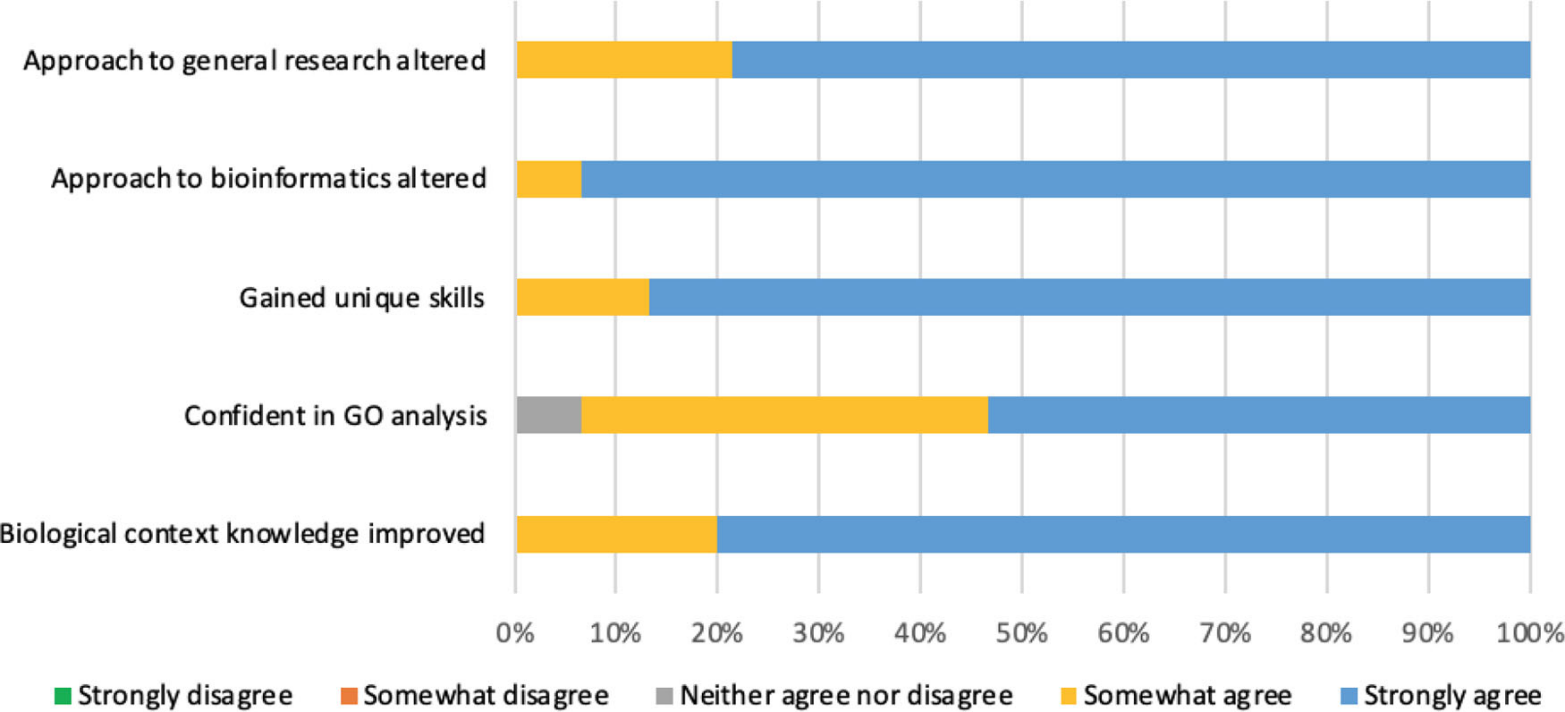
Respondents ranking of learning outcome success. Five questions were included in the survey to assess whether a biocuration project provided the appropriate environment to meet the learning outcome aims. Respondents were asked whether they strongly disagree, somewhat disagree, neither agree nor disagree, somewhat agree or strongly agree that they had achieved each of the learning outcomes of the project.

Five learning outcomes were investigated: improved knowledge of the biological context of the project; unique skills gained; increased confidence in interpretation of GO analyses; altered approach to both general and bioinformatics research. Almost all responses (98.6%) either somewhat or strongly agreed that they had achieved these learning outcomes ( Figure 9). Responses were statistically significant, with Chi ^2^ values ranging from 6.0 × 10 ^−26^ for confidence in GO analysis to 7.63 × 10 ^−72^ for altered approach to bioinformatics research (Table S6). All but one of the respondents (93.3%) strongly agreed that their appreciation of bioinformatics and biocuration increased after undertaking an annotation project ( Figure 9), with one respondent (3446071) stating *“I never knew how important bioinformatics was until after this project”* (Table S1B, Question 12). The individual who answered ‘somewhat agree’ (3432165) to this question also stated that “ *Having acquired some knowledge about bioinformatics through the project, I feel I might be able to use it more efficiently and effectively in future research projects*”, which suggests their response may be down to a lack of confidence rather than a lack of skills, supported by the fact they also answered ‘somewhat agree’ in their confidence in GO analysis. The majority of respondents (78.6%) strongly agreed that their conduction of general scientific research was also changed as a result of their projects (p = 5.88 × 10 ^−49^) with one student stating “… *any future research I conduct will ALWAYS state very clearly the species of all entities utilised!*” (3436438). The submitted statements suggest this behaviour change was due to a greater appreciation of bioinformatics by the students: *“This project definitely helped me have a clearer understanding of what bioinformatics is and the plentiful tools available. This made me appreciate bioinformatics more and the potential and power it has”* (3440328), *“I never knew how important bioinformatics was until after this project*” (3446071, Table S1B, Question 12).

### Themes identified in respondents’ statements

As only 18 students completed the survey there was a limited amount of information that could be gained from the answers provided in the comment fields. However, the respondents’ statements provided a clear consensus about the value of these projects. In addition, these responses covered a very broad range of issues, which provided some interesting insights about the respondents’ view of their annotation projects (Table S9). Only one respondent did not provide any accompanying comments (Table S1B).

Key themes were identified in the student responses provided in all the available comment boxes and coded phrases or “meaning units” were assigned to each theme ( Table 2). The meaning units were then summarised using the text condensation methodology ^34^ (Table S9). The theme with the highest number of meaning units was ‘relevant new skills’ (n = 31) followed by appreciation (n = 19). Each theme had meaning units coded to it from multiple participants, with a range of 29.4% of participants mentioning effective use of databases and project themes to 71% of participants mentioning relevant new skills and newfound appreciation of bioinformatics ( Table 2).

**Table 2.** Key themes identified in student responses. Comments provided in response to the survey ( *Underlying data:* Table S9 ^46^) were combined to identify key themes. Meaning units are extracted phrases from the survey responses. These meaning units were coded to themes identified through initial text analysis of all qualitative responses. The number of respondents whose comments were coded to each theme is shown in brackets.

Theme	Number of meaning units (individual respondents)	Example statements
Relevant new skills	31 (12)	A bioinformatics project would teach me skills that I would be able to apply more readily, regardless of any specialism in a particular area
Appreciation of bioinformatics	19 (12)	I realised the potential of bioinformatics, before I knew that this was a relevant and current area of research but now I fully understand why it is so important and I am interested in looking into bioinformatics in the future as well
Critical analysis and literature review	9 (8)	Improving my ability to critically evaluate papers was crucial in my personal development as it allowed me to widen my understanding of different topics
Excellent support and supervision	9 (8)	Learning new things with excellent supervisors and support
Satisfaction	9 (7)	It was really satisfying to see my annotations get uploaded onto the database
Effective use of databases	7 (5)	Learning how to use the databases and tools with a specific goal in mind makes it much easier to understand and get to grips with and presents a great opportunity to learn from leaders
Enjoyment	7 (6)	I thoroughly enjoyed my bioinformatic dissertation
Accessibility	6 (6)	Data entry can be boring but the thought that it would be **made available and the information could be valuable to researchers** was nice and makes it worthwhile
Project focus	6 (5)	The project was on Alzheimer’s disease, which I was interested in learning about more

An unexpected finding was that almost all of our participants stated they would be interested in participating in a community biocuration initiative (Table S1B, Question 15). Motivation was to both continue implementing their acquired biocuration skills ( *“I think it is important to continue to work on my skills I have gained so far, so being able to be involved in opportunities like these would be very beneficial*”, 3445442) and to contribute to the GO (“ *I believe community biocuration initiative can promote biocuration and in turn aid in improving under represented areas in the ontology. At the same, the community biocuration initiative can aid the incorporation of biocuration in university/research institution settings*”, 3445942).

## Discussion

This survey of students that had either completed or were currently undertaking a GO biocuration project confirmed the value of these assignments. The primary research question, whether GO biocuration projects provide useful and relevant skills to students, was overwhelmingly positive. Additionally, the majority of tasks were considered to be enjoyable as well as useful. The secondary research question addressed whether GO biocuration projects provide a greater overall appreciation and understanding of bioinformatics applications. This was confirmed to be the case, with the majority of participants (80%) confirming that their views of bioinformatics had changed.

### Benefits of biocuration training for students

The number of respondents to our survey (n = 18), limits the interpretation of this data, however the responses are in agreement with those of other similar studies investigating the usefulness of biocuration student projects. ^11^ Hosmani *et al.* (2019), ^11^ discuss the benefits to students undertaking manual annotation efforts including the development of a better understanding of genomics data, the retention of the students in the sciences, and their inclusion on peer-reviewed publications. Our study did not specifically investigate student retention, however all of the respondents to date are still associated with a scientific or medical role.

While it is clear that undertaking a biocuration project leads to the acquisition of general skills that are transferable to other areas of science ^10^^,^^11^ all of our survey respondents appreciated the future benefit of using bioinformatics resources in a research project setting, with one student explaining that *“The experience was also useful for making me aware of future possibilities in terms of where I could apply this knowledge”* (3445442). Manual gene annotation involves more than simply copying and pasting information from one resource to another. ^10^ The biocurator has to learn how to use a variety of bioinformatics resources, understand how to recognise the appropriate gene identifiers, as synonyms are often included in articles rather than approved symbols, and understand how to select the appropriate ontology term for the data presented in an article. ^35^ The advantage of annotation projects is that they provide an environment where several resources are used on a regular basis over an extended period of three to six months. Thus, the students have the opportunity to investigate the range of facilities these resources offer. In addition, a biocurator needs to understand not only the topic being curated, but also the experimental approaches being taken and have the confidence to reject data that does not have sufficient statistical evidence to curate. Thus, the complexity of manual curation provides an opportunity for students to gain experience in applying a variety of bioinformatics resources to answer a research question while gaining a deeper understanding of a specific biological area of interest. ^10^ These annotation projects, therefore, provide unique, in-depth training in multiple bioinformatics resources in a research context. This contrasts with short courses that tend to focus on one particular tool or database rather than how they can be utilized in concert to answer research questions.

It has been well documented that active teaching methods, which require students to use a more problem-based approach, are best used for transference of theoretical knowledge into practical utilisation. ^36^^–^^38^ As biocuration projects provide a supportive active learning environment, it is unsurprising that this study has demonstrated that these projects enable students to consolidate their theoretical knowledge of bioinformatics resources and understand how these can be used in a research setting. However, the success of active teaching methods also heavily depends upon the quality of the supervision. ^39^ In this survey, one of the qualitative themes identified was the excellent level of support and supervision for these projects (Table S9). The students are provided with prompt feedback on the annotations they suggest, and they have weekly one-to-one or small groups meetings with expert biocurators. As such, each of these projects requires a considerable investment of time by expert curators, time which is not always justified by the curation output of the students. Peer review of the annotations by the students themselves would provide a more efficient biocuration project model, and this has been achieved, by a group at Texas A&M, through the establishment of an annotation competition. ^12^ As significant resources are needed to train new biocurators before they are able to work independently, ^29^ biocuration projects, such as the ones provided at UCL, provide substantial initial training for students as biocurators, Indeed, three of our respondents are currently working as biocurators after completion of their projects.

In further agreement with Hosmani *et al*. (2019), ^11^ our survey also confirmed that all participants (whether undergraduate or postgraduate) felt that their biocuration project provided them with the opportunity to increase their understanding of a variety of experimental methods in addition to increasing their appreciation of bioinformatics resources. In addition, for many of the students these projects provided an opportunity to increase their understanding of a biological domain that they intended to research in their future careers. This suggests that biocuration projects could be used to capture the knowledge a new student acquires from their own literature review at the start of their PhD project. Our survey compliments that of other student curation projects and confirms that early research scientists are able to contribute to manual curation efforts to improve existing resources. ^10^^,^^11^ However, an important aspect of these student projects is the curation topic and the need for clear annotation rules or guidelines. Over the past 10 years at UCL we have identified that the students are more comfortable curating microRNAs, for which we have very strict guidelines, than the role of proteins within complex signalling pathways, for which the GO Consortium guidelines are less well defined.

### Community biocuration

One of the biggest challenges to biocuration is the time-consuming nature of reviewing, analysing and organising vast amounts of existing biological knowledge, which creates a bottleneck in genomics research when coupled with limited funding for biocurator roles and lack of trainers. ^10^^,^^29^ Furthermore, the technological advances that have occurred, and are continuing to occur, in the post-genomic era are generating a considerable volume of new data. Consequently, there is an ongoing need to ensure accurate and efficient integration of this data into open-access databases, so that it can be fully exploited. However, there is also a need to integrate well established knowledge, across all areas of biology and biomedicine, into these databases.

One of the ways the biocuration community is trying to increase the volume of curated data is through community biocuration initiatives. ^8- 11^^,^^29^^,^^40^ Annotation projects have the potential to recruit early career researchers to community curation roles. This was confirmed by our survey, with all respondents stating they would be interested in participating in a community biocuration initiative. Primarily our respondents were keen for an opportunity to utilise newly gained skills and continue their contribution to the GO. However, several students were motivated to volunteer to participate in future curation projects because of the satisfaction of knowing that their annotations were being shared and would be of benefit to the wider scientific community. Unfortunately, there are only a very few examples of successful community biocuration efforts ^8^^,^^10^^,^^12^^,^^41^^,^^42^ and, therefore, the opportunities for the wider scientific community to contribute to this need are limited. One of the rate limiting factors in community curation is checking the accuracy of the submitted annotations by expert biocurators. The CACAO competition model has addressed this issue by ensuring annotations are first peer reviewed by other competitors, which reduces the number of errors that need to be corrected before the data is incorporated into a public database. ^12^ As well as benefiting the individual, through continued professional development, community biocuration would result in an enriched coverage of the literature benefiting the wider research community. Another potential source of community curators is retired scientists. These experts may be willing to curate their own articles as well as other seminal articles describing the role of key gene products in their area of expertise.

It is essential for any database to have both a high level of data coverage and accuracy, which requires continuous, high-quality contributions, in order to facilitate discovery. Without integration of raw data and new knowledge into these resources, the discoverability and re-use of this data would be impaired. ^8^ Another challenge for data integration is that many published articles cannot be curated due to a lack of sufficient specific data, which is detrimental to curation efficiency. These biocuration projects alter the students’ perspective of annotation and this could lead to their future research being written with biocurators in mind. Manual curation is a key approach by which databases are able to enhance and remain relevant to investigators and as such is a critical part of scientific work. ^43^ Indeed, as the volume and breadth of data continues to increase, with advancements in both experimental methods and analysis techniques, curated data is fast becoming an essential resource in biomedical research. ^10^^,^^29^

## Conclusion

There is now accumulating evidence that student biocuration projects not only promote better understanding of molecular and cellular biology but can also contribute valuable high-quality annotations that improve existing resources. ^9- 12^ All of the respondents would recommend this project to future students, regardless of study stage or intended future career with respondent 3445942 stating: *“I would highly recommend bioinformatics-based projects for students. It’s an area that is growing tremendously leading to many different career paths in life sciences. It is an important component that is found in any genetics data analysis field.*” It is clear from our data that students gain substantial benefits by undertaking biocuration projects, ranging from improving their ability to carry out literature reviews and critically analyse articles, and extending their understanding of their chosen biological research area, to learning how to exploit bioinformatics resources. Furthermore, these projects lead to an increased appreciation and understanding of the essential and growing role of bioinformatics in scientific investigation. These biocuration projects, therefore, substantially contribute to the student’s overall education.

Annotation projects also benefit the wider biocuration community through a substantial increase in the number of annotations; a total of 5209 annotations have been added to 874 entities following students reviewing 529 articles. Additionally, the students provide a cohort with experience of biocurators who are willing and able to add to the GO knowledgebase throughout their careers through community biocuration initiatives. The importance of continued biocuration cannot be understated and as such, training of potential future bioinformaticians and curators should be a priority for any institution.

## Data availability

### Underlying data

Figshare: Student biocuration projects as a learning environment.xlsx, https://doi.org/10.6084/m9.figshare.16629043.v2. ^46^

This project contains the following underlying data:

- Table S1B: Raw data responses from participants to survey questions.
- Table S2: Summary of participant demographics.
- Table S3: Summary of responses to usefulness and enjoyability of general (blue) and specific (green) project aspects.
- Table S8: Student negative responses to biocuration project (Questions 7 and 8).
- Table S9: Meaning Units assigned to each qualitative theme identified through text condensation methodology.

### Extended data

Figshare: Student biocuration projects as a learning environment.xlsx, https://doi.org/10.6084/m9.figshare.16629043.v2. ^46^

This project contains the following extended data:

- Table S1A: Questions included in the Opinio survey, with format of answer options and coding associated with specific answers.
- Table S4: F-tests carried out for usefulness and enjoyability of general and specific aspect variance as well as prior and post bioinformatics knowledge with or without attendance of the GHD bioinformatics module.
- Table S5: t-tests for comparison of usefulness and enjoyability of general and specific project aspects as well as prior and post-project bioinformatics knowledge with or without attendance of the GHD bioinformatics module.
- Table S6: Chi2 tests for comparison of expected and observed responses to usefulness and enjoyability of project aspects as well as learning outcomes.
- Table S7: ANOVA comparison of means for prior and post-project bioinformatics knowledge and attendance of GHD bioinformatics module.
- Table S10: List of activities undertaken by the student and supervisor during a student GO annotation project.

Data are available under the terms of the Creative Commons Attribution 4.0 International license (CC-BY 4.0).
