# Supplementary material for: Student biocuration projects as a learning environment
Source: F1000Res. 2022 Feb 2;10:1023. Originally published 2021 Oct 8. [Version 2] doi: 10.12688/f1000research.72808.2 (PMC8831850; doi:10.12688/f1000research.72808.2)
Supplement: Peer review history

# Peer review: Reviewer response for version 2

**DOI:** 10.5256/f1000research.119256.r121941

**Published:** 2022-02-10

**Authors:** Anna Delprato

**Affiliations:** 1 Department of Research and Education, BioScience Project, Wakefield, MA, USA

The authors have addressed my suggested edits in the revised article. I have no further comments.

Is the work clearly and accurately presented and does it cite the current literature?

Yes

If applicable, is the statistical analysis and its interpretation appropriate?

Yes

Are all the source data underlying the results available to ensure full reproducibility?

Yes

Is the study design appropriate and is the work technically sound?

Yes

Are the conclusions drawn adequately supported by the results?

Yes

Are sufficient details of methods and analysis provided to allow replication by others?

Partly

Reviewer Expertise:

Systems Genetics

I confirm that I have read this submission and believe that I have an appropriate level of expertise to confirm that it is of an acceptable scientific standard.

# Peer review: Reviewer response for version 1

**DOI:** 10.5256/f1000research.76411.r99639

**Published:** 2021-12-02

**Authors:** Anna Delprato

**Affiliations:** 1 Department of Research and Education, BioScience Project, Wakefield, MA, USA

The article by Thurlow *et al.* describes the utility of implementing a biocuration project to teach students how to critically evaluate primary scientific literature, and at the same time contribute annotations to the Gene Ontology database, which is a valuable resource used by the scientific community worldwide. The authors have provided data evaluating student experience and project enjoyability, as well as learning outcomes. This is an interesting and useful project design, which is clearly beneficial to students and the research community. I have several comments that need to be addressed.

**Introduction:**

- "utilised in a plethora of future careers" - Please indicate which careers these skills could be useful for.
- "Recent technological advancements in high-throughput research have resulted in vast amounts of data output, which is outstripping analysis capabilities" - Please indicate which technological advances specifically? 

**Methods:**

- The authors should explain how the literature searches for annotation are performed.
- How are the articles selected?
- Are students involved in article selection?
- If not, do they learn this as part of the training?
- Are Pubmed, Google Scholar, and MeSH used to find articles for curation?
- What is the criteria for article selection?
- Are the articles mostly current?
- Are the use of certain methods a factor in the selection process?

**Other items:**

- Reference 31 the citation link is broken

**Supplementary Workbook:**

- Table S2. "undertaken" is misspelled in the title.
- Authors should recheck spelling throughout the supplementary workbook..

Is the work clearly and accurately presented and does it cite the current literature?

Yes

If applicable, is the statistical analysis and its interpretation appropriate?

Yes

Are all the source data underlying the results available to ensure full reproducibility?

Yes

Is the study design appropriate and is the work technically sound?

Yes

Are the conclusions drawn adequately supported by the results?

Yes

Are sufficient details of methods and analysis provided to allow replication by others?

Partly

Reviewer Expertise:

Systems Genetics

I confirm that I have read this submission and believe that I have an appropriate level of expertise to confirm that it is of an acceptable scientific standard, however I have significant reservations, as outlined above.

# Response

**Published:** 2021-12-22

**Authors:** Ruth Lovering

**Affiliations:** University College London, UK

We appreciate the suggestions made by Dr Delprato. The revisions we have made to address Dr Delprato concerns are listed below. We hope that these are sufficient to address these points.

**Introduction:**

 1a) "utilised in a plethora of future careers" - Please indicate which careers these skills could be useful for.

**Manuscript revisions**

 In the first paragraph of the introduction, we have provided a short list of potential careers: ‘ *As such, a research project in bioinformatics requires the incorporation of skills from a multitude of areas and can be utilised in a plethora of future careers, such as computational biologists, bench scientists, project managers, copywriters, clinicians, bioinformaticians or administrators.*’

 In addition, we have added a list of transferable skills that students will have developed after completing a biocuration project, at the start of the last paragraph of the introduction: ‘ *Students develop a range of transferable skills during the GO biocuration projects, that they would have developed when undertaking a bench or literature review project. For example, the students improve their writing and communication skills, their* *project and time management skills* *, as well as their ability to* *find and understand relevant data, critically evaluate this data and summarise the information*.’

**Methods:**

- The authors should explain how the literature searches for annotation are performed.
- How are the articles selected?
- Are students involved in article selection?
- If not, do they learn this as part of the training? 
- Are Pubmed, Google Scholar, and MeSH used to find articles for curation?
- What is the criteria for article selection?
- Are the articles mostly current?
- Are the use of certain methods a factor in the selection process?

**Manuscript revisions**

 The method section has been revised and an additional supplementary table created (S10) has been created and the new version of the supplementary tables is available 10.6084/m9.figshare.16629043. This table lists the activities the student and supervisors undertake during the ‘curation’ part of the project.

 In the Methods, Participants and Setting section the following two paragraphs have been added:

*‘All of the participants had undertaken a GO biocuration project, however, while the format for each project was generally the same, there was some variation. These variations were due to the study level of the student (which ranged from undergraduate to post-graduate), the interest of the student in learning key aspects of biocuration, the biological knowledge of the student, the title of the project, the project length (ranging from 4-8 months, full or part-time) and finally the timing of the project with respect to the Covid-19 pandemic. A list of the activities undertaken during the project are provided in Supplementary Table S10.*

*All students chose a specific area of biology, usually linked to a specific disease, to curate. Some of the students focused on curating proteins, while others focused on microRNAs, but all students follow existing GO consortium guidelines ^26,35,47^. For protein focused annotation projects, articles for curation are identified by a PubMed search using HUGO gene nomenclature (HGNC) approved symbols ^48^, and key words (if there are more than 10 articles to review). Articles are only curated if there is sufficient information about the species of the protein used in the experiments and the article provides experimental data that describes the role of the protein in the disease or process that the student is curating. For microRNA projects the HGNC symbol of the microRNA target is used to search either miRTarBase ^49^or emiRIT ^50^. If no suitable articles are identified, then PubMed is searched with the HGNC symbol and key words such as microRNA (Supplementary Table S10). Articles are only curated if there is evidence that the microRNA binds to the target mRNA that encodes for one of the prioritised proteins (usually this is provided by a reporter assay) and there is sufficient information about the species of the microRNA and mRNA used in the experiments. The publication date is not usually used to filter articles to curate.’*

**Other items:**

- Reference 31 the citation link is broken

**Manuscript revision**

 I am unable to edit the citation links. Also the new references added are not linked to the reference list and are also out of sequence.

**Supplementary Workbook:**

- Table S2. "undertaken" is misspelled in the title.  
- Authors should recheck spelling throughout the supplementary workbook.

**Manuscript revision**

 The supplementary tables have been checked and all spelling mistakes corrected. The new version of the supplementary tables is available 10.6084/m9.figshare.16629043.

# Peer review: Reviewer response for version 1

**DOI:** 10.5256/f1000research.76411.r96513

**Published:** 2021-10-27

**Authors:** Nicole A. Vasilevsky

**Affiliations:** 1 Anschutz Medical Campus, University of Colorado, Aurora, CO, USA; 2 Translational and Integrative Sciences Lab, Oregon State University, Corvallis, OR, USA

This article nicely describes student perceptions of a biocuration training course at the University College of London.

 This paper is well written and provides nice examples of the benefits of this course, through the increased GO annotations contributed by the students. The survey results provide evidence for the usefulness and the positive impact of the course.

 In the introduction, it would be great to have a more detailed description of your definition of bioinformatics and its relationship to the biocuration field.

 It would be nice to read about how the students performed in the course. Were they all successful at performing quality annotations or did the quality vary amongst the students? Did it improve over time? If you were to perform a future study on the outcomes of this training, it would be nice to see inter-curator agreement between students and between the student and expert curator/mentor.

 It would be interesting to hear more about the community biocuration effort that was offered as a follow-up to this course.

Is the work clearly and accurately presented and does it cite the current literature?

Yes

If applicable, is the statistical analysis and its interpretation appropriate?

Yes

Are all the source data underlying the results available to ensure full reproducibility?

Yes

Is the study design appropriate and is the work technically sound?

Yes

Are the conclusions drawn adequately supported by the results?

Yes

Are sufficient details of methods and analysis provided to allow replication by others?

Yes

Reviewer Expertise:

Biocuration, biomedical ontology development, ontology, and data management training

I confirm that I have read this submission and believe that I have an appropriate level of expertise to confirm that it is of an acceptable scientific standard.

# Response

**Published:** 2021-10-27

**Authors:** Ruth Lovering

**Affiliations:** University College London, UK

We thank the reviewer for these positive comments and interest in our article. A definition of bioinformatics is challenging, the course we provide the students only touches on this field, the areas covered are all mentioned in the survey. As biocuration provides biological annotations it is certainly is an essential component of many aspects of bioinformatics.  

 The majority of students were able to provide very good annotations for at least 15, but often 30 articles. However, there was considerable variability in how much feedback was required during the project and how quickly the student reached an understanding that enabled them to create high quality annotations. For some students this took around a month, for others it was only towards the end of the project that this was achieved.

 Inter-curator annotation consistency is an interesting discussion point, especially as it can be difficult to achieve this in annotation groups. The number of students undertaking annotation projects at UCL each year prevents this from an area we can investigate; the students are required to submit a dissertation and do not have time to review another student’s annotations. However, Ramsey *et al.* (in press) discuss a long running project which involves students assessing other students’ annotations.

 Only a few students have continued to provide annotations after the end of their annotation project and consequently there is very little to report on this.
